# Antipsychotics-induced improvement of cool executive function in individuals living with schizophrenia

**DOI:** 10.3389/fpsyt.2023.1154011

**Published:** 2023-04-27

**Authors:** Yajing Si, Congcong Liu, Yanna Kou, Zhao Dong, Jiajia Zhang, Juan Wang, Chengbiao Lu, Yanyan Luo, Tianjun Ni, Yunhong Du, Hongxing Zhang

**Affiliations:** ^1^School of Psychology, Xinxiang Medical University, Xinxiang, Henan, China; ^2^Xinxiang Key Lab for Psychopathology and Cognitive Neuroscience, Xinxiang, Henan, China; ^3^Second Affiliated Hospital of Xinxiang Medical University, Xinxiang, Henan, China; ^4^Zhumadian Second People's Hospital, Zhumadian, Henan, China; ^5^Henan International Key Laboratory for Non-invasive Neuromodulation, Xinxiang, Henan, China; ^6^School of Nursing, Xinxiang Medical University, Xinxiang, Henan, China; ^7^School of Basic Medical Sciences, Xinxiang Medical University, Xinxiang, Henan, China

**Keywords:** schizophrenia, prediction, cool executive function, electroencephalogram, brain network

## Abstract

Cool executive dysfunction is a crucial feature in people living with schizophrenia which is related to cognition impairment and the severity of the clinical symptoms. Based on electroencephalogram (EEG), our current study explored the change of brain network under the cool executive tasks in individuals living with schizophrenia before and after atypical antipsychotic treatment (before_TR vs. after_TR). 21 patients with schizophrenia and 24 healthy controls completed the cool executive tasks, involving the Tower of Hanoi Task (THT) and Trail-Marking Test A-B (TMT A-B). The results of this study uncovered that the reaction time of the after_TR group was much shorter than that of the before_TR group in the TMT-A and TMT-B. And the after_TR group showed fewer error numbers in the TMT-B than those of the before_TR group. Concerning the functional network, stronger DMN-like linkages were found in the before_TR group compared to the control group. Finally, we adopted a multiple linear regression model based on the change network properties to predict the patient’s PANSS change ratio. Together, the findings deepened our understanding of cool executive function in individuals living with schizophrenia and might provide physiological information to reliably predict the clinical efficacy of schizophrenia after atypical antipsychotic treatment.

## 1. Introduction

Schizophrenia (SP) is a severe psychiatric disorder, and most individuals living with schizophrenia have a wide range of cognitive impairments (1). Moreover, executive dysfunction has been postulated to be the characteristic neuropsychological symptom of schizophrenia, which indicates that patients could not integrate multiple information and processes efficiently to achieve a required goal (2). Executive functions are divided into hot and cool executive functions: the former involves a high degree of motivational and emotional activation; the latter is seen as a high cognitive process that regulates the input and output stimulus independently to make a behavioral response flexibly (3). The cool executive function is appropriate and objective for exploring the executive function in people living with schizophrenia because it is irrelevant to a person’s emotional arousal, as well as the emotional reactions in patients with SP are usually inconsistent with their inner experiences (4). There is a range of classical cool executive paradigms that are utilized to probe the deficit degree of executive function in patients with SP. For instance, patients with SP showed a slower reaction time and poorer performance in Trail-Marking Test A-B (TMT A-B) which measures a subject’s primitive consciousness movement rates and the cognitive set transfer function (4). Through the Tower of Hanoi task (THT), researchers have explored the individual ability to generate rules and make a plan according to those rules (5), and the findings of a meta-analysis showed a planning deficit in patients with SP which was moderated by task difficulty of the minimum number of moves asked for a solution (6). Given the characteristics of SP and the roles of the task itself, the tasks (i.e., THT and TMT A-B) that are easy to operate are often chosen to explore the cool executive function in patients with SP. The above showed there was damage to cool executive function in patients with SP, mainly involving the insufficiencies in planning, response inhibition, shifting, working memory, and selecting information.

The atypical antipsychotic is the cornerstone of the treatment in people living with SP, in addition to controlling the clinical symptoms, multiple studies have implemented antipsychotic interventions to improve the cognitive abilities in the independent daily life in patients with SP, reflecting its another clinical treatment outcome (7, 8). For example, there were modest improvements in attention and verbal fluency after the clozapine treatment in patients with SP (9). Pardo et.al explored the changes in cognitive flexibility of patients with SP after antipsychotic treatment, and revealed that the treatment improved patients’ cognitive flexibility in the test for attentional performance (10). Expect for cognitive flexibility, aripiprazole has been revealed to improve patients’ reaction time with correct responses to task current stimuli (11). While patients with SP performed the Wisconsin Card Sorting Test (WCST) after an 8-week antipsychotics treatment, there was significantly improved perseverative, and a trend for WCST categories improvement was observed (12). Taken together, the cognitive function of patients with SP could be improved by antipsychotic treatment interventions. Besides the use of antipsychotics, to improve the durability and generalization of the treatment, the cognitive remediation of the psychosocial intervention was used to improve cognitive function in people living with SP. The most recent meta-analysis with 130 studies revealed a consistent small-to-moderate positive effect of cognitive remediation treatment for individuals living with SP (13), which was in line with the results observed in another methodologically rigorous meta-analysis (14). Although psychosocial intervention, physical exercise, and noninvasive brain stimulation are valid for treatment in people living with SP, antipsychotic intervention presents substantial benefits on clinical symptoms and is the cornerstone of clinical treatment.

Nevertheless, the changes in the brain networks in cool executive function before and after antipsychotic intervention in patients with SP are still left unveiled. Electroencephalogram (EEG) (15–17) has been widely utilized to reveal the underlying neural mechanism during the cognitive process, such as attention, working memory, and decision-making. A typical brain network of EEG involves many related brain areas, and the information is processed efficiently within the network through the functionally linked areas. Four weighted network properties (18), involving clustering coefficient (*CLU*), local efficiency (*LE*), global efficiency (*GE*), and characteristic path length (*L*), corresponding to the weighted network were utilized to evaluate the EEG network synchronization toward the patients in SP. Based on the network analysis, the cognitive deficits were revealed in people living with SP, showing top-down dysconnectivity during P300 tasks (19). Therefore, based on the three classical cool executive tasks (THT, TMT A-B), we adopted the brain network analysis of EEG in the current research to investigate the changes in brain network in patients with SP before and after atypical antipsychotic treatment for 8 weeks (before_TR vs. after_TR). We also explored the potential relationship between the symptom change index and the change of brain network properties during the process of cool executive tasks before and after treatment, from which a model was built to predict the clinical treatment efficacy in people living with SP.

## 2. Materials and methods

### 2.1. Participants

Twenty-one patients with SP were recruited from inpatient and outpatient psychiatric units at the Second Affiliated Hospital of Xinxiang Medical University from December 2014 to May 2015. The whole experiment was conducted in the lab of the Second Affiliated Hospital of Xinxiang Medical University. The inclusion criteria for patients with SP were that: (1) subjects had been diagnosed with schizophrenia by two experienced psychiatrists utilizing Structured Clinical Interview for the DSM-IV Axis I Disorder, patient edition (SCID-I/P); (2) they were between 18 and 40 years old, and right-handed; and (3) they could respond and understand relevant rules properly. Twenty-four right-handed healthy controls (HC) were recruited from the community, with the inclusion criteria of the absence of a current or lifetime diagnosis of Axis I or II disorders. Exclusion criteria for both groups were: (1) they were diagnosed with a neurological illness; (2) they had language, visual, or auditory deficits; and (3) they had alcohol or drug abuse/dependence within 3 months.

The two groups were matched for sex, age, and years of education. Detailed information could be found in Table 1. Written informed consent was obtained from all participants before taking part in our experiment according to the Declaration of Helsinki. The current study was approved by the Ethics Committee of Xinxiang Medical University.

**Table 1:** Demographic data information for the schizophrenia group (SP) and the healthy control group (HC).

Group	*N*	Sex (M/F)	Age, years (range)	Education, years (range)	Illness duration, years (range)
SP	21	9/12	27.86 ± 7.64	13.49 ± 2.30	5.49 ± 5.83
HC	24	14/10	23.83 ± 3.89	13.85 ± 3.04	N/A

### 2.2. Experimental procedures

#### 2.2.1. Clinical evaluation

Subjects’ clinical symptoms were evaluated by the positive and negative syndrome scale (PANSS) with 30-item which included three factors, such as positive symptoms (7 items), negative symptoms (7 items), and general psychopathology (16 items). Subjects’ information was measured in the week prior to the evaluation. The evaluation time is 30–50 min for each subject. The reliability and validity of PANSS were tested by Kostakoglu et al. in 1999 (20). The scale was assessed by trained psychiatrists. Then, the patient’s PANSS change ratio was obtained. And the PANSS change ratio is the pre-treatment score minus the post-treatment score and then divided by the pre-treatment score.

#### 2.2.2. Intervention

The patients with SP had not taken medication for more than 3 months at the time of initial enrollment and were then taking atypical antipsychotics. In detail, there were 10 patients with 4-6 mg risperidone, 8 patients with 20–30 mg olanzapine, and 3 patients with 15–30 mg aripiprazole per day. Antipsychotics doses increased to a standard therapeutic range within 1 week. During the current study, no other antipsychotics were utilized.

#### 2.2.3. Cool execution tasks

**Figure 1:**
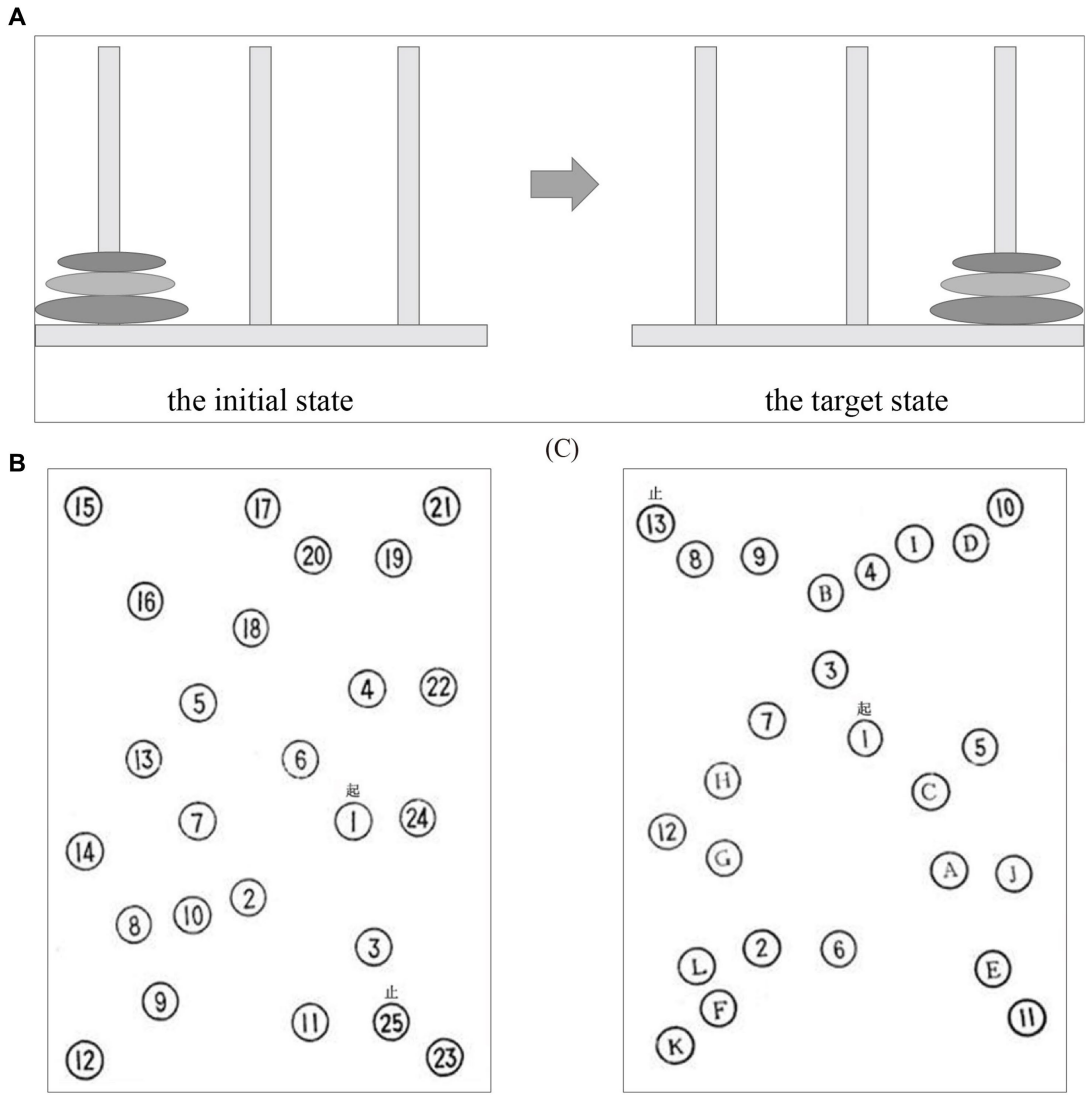
The three cool executive tasks: **(A)** the Tower of Hanoi Task; **(B)** Trail-Marking Test A; **(C)** Trail-Marking Test B.

After the initial enrollment, all subjects conducted the EEG experiment that consisted of three cool executive tasks, involving the THT and TMT A-B. In the THT task (Figure 1), there were three tower bases, respectively, that were corresponding to three wood blocks with distinct diameters, and the subject was instructed to move the wood blocks from the initial location to the target location following three rules: a. one block must be moved only one step; b. the block needed to be placed on one of the three tower bases or the subject’s hand; c. the larger block could not be presented on the top of the small block. The reaction time and the number of operative steps were utilized to evaluate the ability of individual cool execution function in the THT. In the TMT-A, the subjects needed to link the numbers from 1 to 25 as quickly as they could with a pen, and the pen point must remain in contact with the paper during the processing. The subject was guided to link numbers (i.e., 1–13) and letters (i.e., A-L) quickly based on an alternating sequence in TMT-B. The reaction time and error numbers could be obtained to assess the performance of the subject in the two tasks (Figures 1B,C). The subjects were offered practices to ensure the rules of the three cool executive tasks were understood, respectively, before the formal experiment began.

### 2.3. EEG data acquisition

Through the Cerebus 128TM Amplifier (Cyberkinetics In, American), the EEG dataset with 64 Ag/AgCl electrodes from the 10–20 system was acquired (Supplementary Figure S1). The raw EEG was filtered with 0–250 Hz, and the sampling rate was 1000 Hz. The reference and ground electrodes were REF and GND, respectively. The impedance of all electrodes was kept below 5 kΩ in the whole EEG experiment. During the processing of the resting-state EEG acquisition, the participants were asked to close their eyes and sat in a comfortable position, staying as quiet and relaxed as possible in a shielded room. Combined with a 5-min resting-state EEG, 2-min break, and task-related EEG, there were about 12-min during the EEG acquisition.

### 2.4. EEG data processing

#### 2.4.1. EEG pre-processing

A crucial problem for EEG analysis is that EEG signals are susceptible to physiological artifacts during the processing of data recording, especially ocular artifacts. In this study, we adopted an independent component analysis (ICA) algorithm and wavelet denoising to separate the ocular artifacts from our EEG data: (1) The discrete wavelet transform decomposition of the artifacts ingredients was measured. (2) The threshold *K* was defined, and we judged the wavelet coefficient, i.e., if the coefficient exceeded the threshold, it was set to 0. The constant was 0.6745, which was the wideband neuronal signal for Gaussian noise. (3) The inverse discrete wavelet transform of the thresholded coefficients was evaluated, and the EEG by inverse-transformed wavelet coefficients was rebuilt. The subsequent pre-processing procedures, involving averaging referencing, 0.1–45 Hz band-pass filtering, the 3-s-length data segmenting, and artifact trial removal (±120 μV serve as threshold), were utilized in the current study. After the task-related EEG data preprocessing, the number of remaining segments (trials) was 40 ± 29 for the before_TR group, 20 ± 13 for the HC group, and 33 ± 22 for the after_TR group.

#### 2.4.2. EEG network analysis

Next, coherence (COH) was used to construct the corresponding network for each segment from the EEGs of the three cool executive tasks. Finally, the individual adjacency matrix was averaged across all segments to estimate the task-related connectivity matrix. We used the 21 canonical electrodes of 10–20 international system in this study, involving FP1, FP2, FPz, F7, F3, F4, Fz, F8, T7, C3, C4, Cz, T8, P7, P3, P4, Pz, P8, O1, O2, and Oz, to reduce the effect of volume conduction during the network analysis (21, 22). Based on the 3-s EEG segments after the preprocessing procedure, we utilized the coherence to evaluate the functional connectivity and the synchrony-defined neuronal assemblies at each frequency point between every pair of electrodes (18). Coherence is formulated as follows,

(1) $$C_{XY}\left(f\right)=\frac{{\left|P_{XY}\left(f\right)\right|}^{2}}{P_{XX}\left(f\right)P_{YY}\left(f\right)}$$

where *P_XY_(f)* is the cross-spectrum of *X(t)* and *Y(t)* signals at the frequency point *f*. In addition, *P_XX_(f)* and *P_YY_(f)* express the auto spectrum assessed from the Welch-based spectrum of *X(t)* and *Y(t)*. *C_XY_(f)* indicates the frequency-dependent coherence. The network edge was estimated by averaging the coherence value over the interested frequency range, *C_XY_*, which conducted 21 × 21 weighted adjacency matrices for every segment. By averaging the individual adjacency matrices of the artifact-free segments, we obtained a final brain network that worked on EEG in subjects with different cool executive tasks.

To quantitatively estimate the brain network while processing related cool executive information, four network properties in the current study, namely, *CLU*, *LE*, *GE*, and *L* were calculated. *C_ij_* is the coherence value between *i* and *j*. *N* expresses the node number, *θ* denotes the set of all nodes in a given brain network, and *d_ij_* indicates the shortest path length between *i* and *j*. These four network properties are expressed as follows,

(2) $$CLU=\frac{1}{N}\underset{i\in \theta }{\sum }\frac{\sum_{j,l\in \theta }{\left(C_{ij}C_{il}C_{jj}\right)}^{1/3}}{\sum_{j\in w_{ij}}\left(\sum_{j\in \theta }C_{ij}-1\right)}$$

(3) $$LE=\frac{1}{N}\underset{i\in \theta }{\sum }\frac{\sum_{j,l\in \theta ,j\neq i}{\left(C_{ij}C_{il}{\left[d_{jl}\left(\theta_{i}\right)\right]}^{-1}\right)}^{1/3}}{\sum_{j\in \theta }C_{ij}\left(\sum_{j\in \theta }C_{ij}-1\right)}$$

(4) $$GE=\frac{1}{N}\underset{i\in \theta }{\sum }\frac{\sum_{j\in \theta ,j\neq i}d_{ij}{}^{-1}}{N-1}$$

(5) $$L=\frac{1}{N}\underset{i\in \theta }{\sum }L_{i}=\frac{1}{N}\underset{i\in \theta }{\sum }\frac{\sum_{j\in \theta ,j\neq i}d_{ij}}{N-1}$$

We adopted the brain connectivity toolbox (BCT)^1^ to measure the four brain network properties for the three cool executive networks in our study.

### 2.5. Statistical analysis

To build a prediction model of the clinical efficacy (PANSS change ratio) of the patients with SP, the four change network properties (i.e., *CLU*, *LE*, *GE,* and *L*) before and after treatment were utilized as the variables in the multiple linear regression model. First, the collinearity diagnostics were used (the variance inflation factor < 10 and the tolerance >0.1), and the multiple linear regression model was built subsequently. We adopted the leave-one-out cross-validation strategy in the current study to predict the clinical efficacy in patients with SP (23). The leave-one-out cross-validation is the leave-one subject out. In each cross-validation, *n*-1 samples were used for training, and the remaining 1 sample was used for testing. Subsequently, we assessed the regression coefficient for the four variables, and the prediction model for *n*-1 samples was built. Then, we adopted the prediction model to predict the PANSS change ratio of a subject in the test set. In this study, the above procedures were repeated *n* times until all subjects served as a testing set for one time. To evaluate the prediction performance, we then obtained the correlation coefficient between the predicted and actual PANSS change ratios through the Person’s correlation analysis with a false discovery rate (FDR) correction. Finally, we adopted the root mean square error (RMSE) to estimate the prediction error. A smaller RMSE corresponds to a better prediction of clinical efficacy in people living with SP.

The SPSS statistics 17.0 was utilized in the current work to explore the cool executive function differences in the patients with SP before and after atypical antipsychotic treatment. The Shapiro–Wilk test was utilized to assess the distribution of data. The independent *t*-test was used to quantify the differences in behaviors and brain networks following normal distribution in the three cool executive tasks between the before_TR group and the HC group. The paired *t*-test was used to quantify the differences (i.e., behaviors, PANSS scores, and brain networks) following normal distribution in the three cool executive tasks between the before_TR and after_TR groups. Non-parametric tests were used to explore the difference in the number of operative steps and errors which were not fit the normal distribution in tasks among different groups. During the statistical analysis, a significant level of 0.05 was set, if the value of *p* is smaller than 0.05, the result then shows a significant between-condition difference. We also explored the potential relationships between the symptom change index and the change of brain network properties during the process of cool executive tasks before and after treatment, from which a model was built to predict clinical efficacy in people living with SP. Pearson’s correlation with an FDR correction was adopted to explore the relationship between cognitive performance and PANSS in pre-treatment patients.

## 3. Results

### 3.1. The before_TR group vs. the HC group

We explored the behavioral difference between the before_TR group and the HC group during the three cool executive tasks (i.e., THT, TMT-A, and TMT-B). In the THT, there was no significant difference between the two groups in the reaction time and the operative step (*p* > 0.05). We found that the reaction time of the before_TR group was much longer (*p* < 0.001) than that of the HC group in the TMT-A, while there was no significant difference in the error number between the two groups (*p* > 0.05). In the TMT-B, compared to the HC group, the before_TR group showed a longer reaction time (*p* < 0.001) and more error numbers (*p* < 0.001; Table 2). Through Pearson’s correlation analysis between the cool executive tasks and the clinical symptoms in the before_TR group, we found that there was a positive relationship between the reaction time in TMT-A and the patients’ positive symptoms (*r* = 0.50; *p* = 0.02).

**Table 2:** Behavioral indicators in the cool executive tasks for the before_TR group and the control group.

		Group		*U*/*t*
		before_TR (*n* = 21)	Control (*n* = 24)	
Task 1	RT	90.71 ± 61.17	66.42 ± 52.03	1.44
	Step	18.38 ± 11.40	14.17 ± 7.53	200.50
Task 2	RT	63.57 ± 22.96	35.29 ± 10.35	5.20^**^
	Error	0.38 ± 0.81	0.29 ± 0.62	243.50
Task 3	RT	203.00 ± 94.05	82.33 ± 35.16	5.55^**^
	Error	4.19 ± 4.33	0.33 ± 0.82	118.50^**^

*p < 0.05, **p < 0.001. RT: the reaction time; Step: the operative step; Error: the error number.

**Figure 2:**
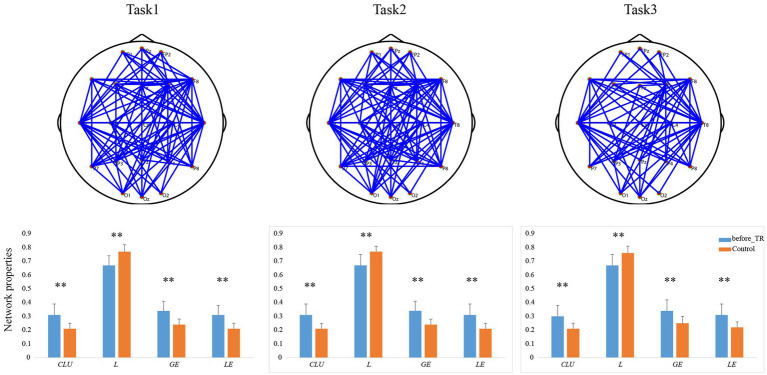
The first line is network topology differences between the before_TR group and the control group in the cool executive tasks (Task 1: THT; Task 2: TMT-A; Task 3: TMT-B). The corresponding network properties in the three tasks were below the network topologies. ** indicates *p* < 0.01. The blue solid lines denote the stronger network edges (*p* < 0.05, FDR corrected) of the before_TR group compared to those of the control group.

Based on the coherence brain network analysis, we further explored the underlying network differences between the before_TR group and the HC group in the cool executive tasks (THT; TMT-A; TMT-B) by using an independent-sample *t*-test. In Figure 2, the EEG-based default mode network (DMN) exhibits stronger activity in the before_TR group compared to that of the control group in the cool executive tasks (*p* < 0.05, FDR corrected). We quantitatively measured the corresponding network properties by four measurements (*Clu*, *Le*, *Ge,* and *L*), as well as the underlying differences between the before_TR group and the HC group. As illustrated in Figure 2, increased *Clu*, *Ge*, and *Le*, as well as a shorter *L* in the three tasks, were observed in the before_TR group (*p* < 0.05).

### 3.2. The before_TR group vs. the after_TR group

**Figure 3:**
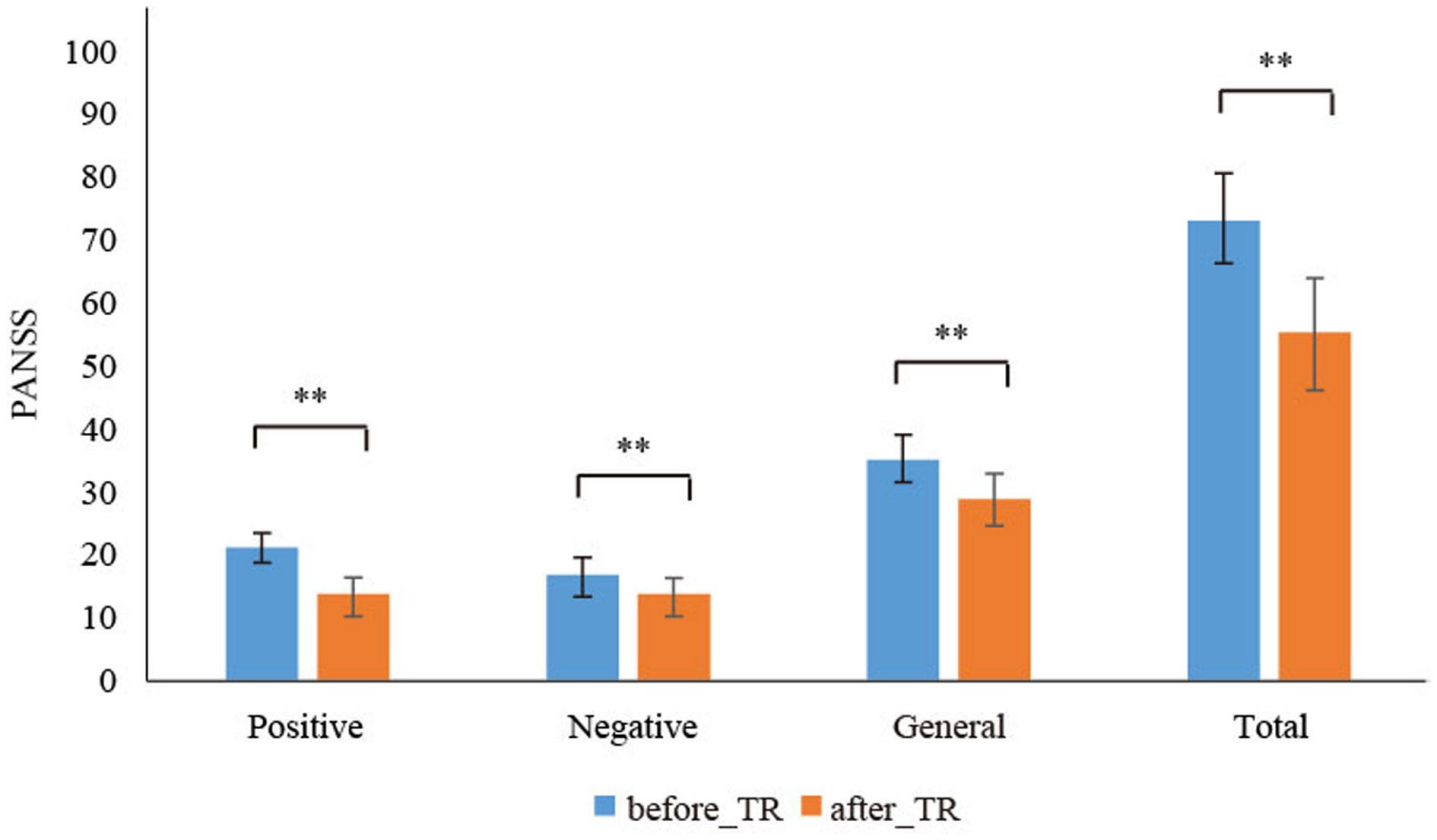
The PANSS scores for the before_TR group and the after_TR group. ** indicates *p* < 0.01.

Table 3 displays that, in the TMT-A and TMT-B, we found that the reaction time of the after_TR group was much shorter than that of the before_TR group (*p* < 0.05). There was no significant difference in the operative step in THT and the error number in TMT-A between the two groups. In the TMT-B, compared to the before_TR group, the after_TR group showed fewer error numbers (*p* = 0.04). Figure 3 shows improvement in symptoms in patients with SP after treatment. Three PANSS symptoms, namely the positive and negative symptoms, and the general psychopathological, and the total PANSS score showed significant improvement in the after_TR group (21.38 ± 3.63; 17.57 ± 5.24; 35.67 ± 6.83; 74.62 ± 13.76) relative to those of the before_TR group (14.29 ± 5.43; 14.29 ± 5.25; 28.67 ± 7.96; 57.24 ± 17.40) (*p* < 0.001).

**Table 3:** Behavioral indicators in the cool executive tasks for the before_TR group and the after_TR group.

		Group		Z/*t*
		before_TR (*n* = 21)	after_TR (*n* = 21)	
Task 1	RT	90.71 ± 61.17	87.67 ± 61.27	0.20
	Step	18.38 ± 11.40	19.38 ± 9.88	−0.24
Task 2	RT	63.57 ± 22.96	50.57 ± 18.93	4.41^**^
	Error	0.38 ± 0.81	0.45 ± 1.15	−0.14
Task 3	RT	203.00 ± 94.05	160.95 ± 56.72	2.42^*^
	Error	4.19 ± 4.33	2.00 ± 1.92	−2.07^*^

*p < 0.05, **p < 0.001. RT: the reaction time; Step: the operative step; Error: the error number.

**Figure 4:**
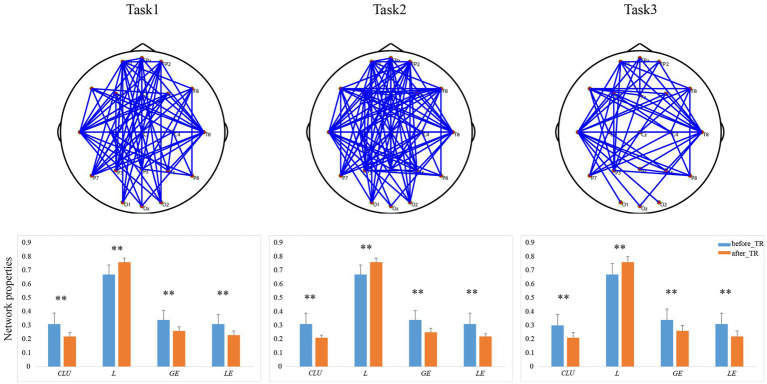
The first line is network topology differences between the before_TR group and the after_TR group in the cool executive tasks (Task 1: THT; Task 2: TMT-A; Task 3: TMT-B). The corresponding network properties in the three tasks were below the network topologies. ** indicates *p* < 0.01. The blue solid lines denote the stronger network edges (*p* < 0.05, FDR corrected) of the before_TR group compared to those of the after_TR group.

We further explored the underlying network differences between the before_TR group and the after_TR group in the cool executive tasks (THT; TMT-A; TMT-B). In Figure 4, the EEG-based DMN exhibits stronger activity in the before_TR group compared to that of the after_TR group in the cool executive tasks (*p* < 0.05, FDR corrected). We quantitatively analyzed the differences between the before_TR group and the after_TR group in the four network measurements. As illustrated in Figure 4, increased *CLU*, *LE*, *GE*, as well as a shorter *L* in the three tasks, were observed in the before_TR group (*p* < 0.05).

### 3.3. The after_TR group vs. the HC group

**Figure 5:**
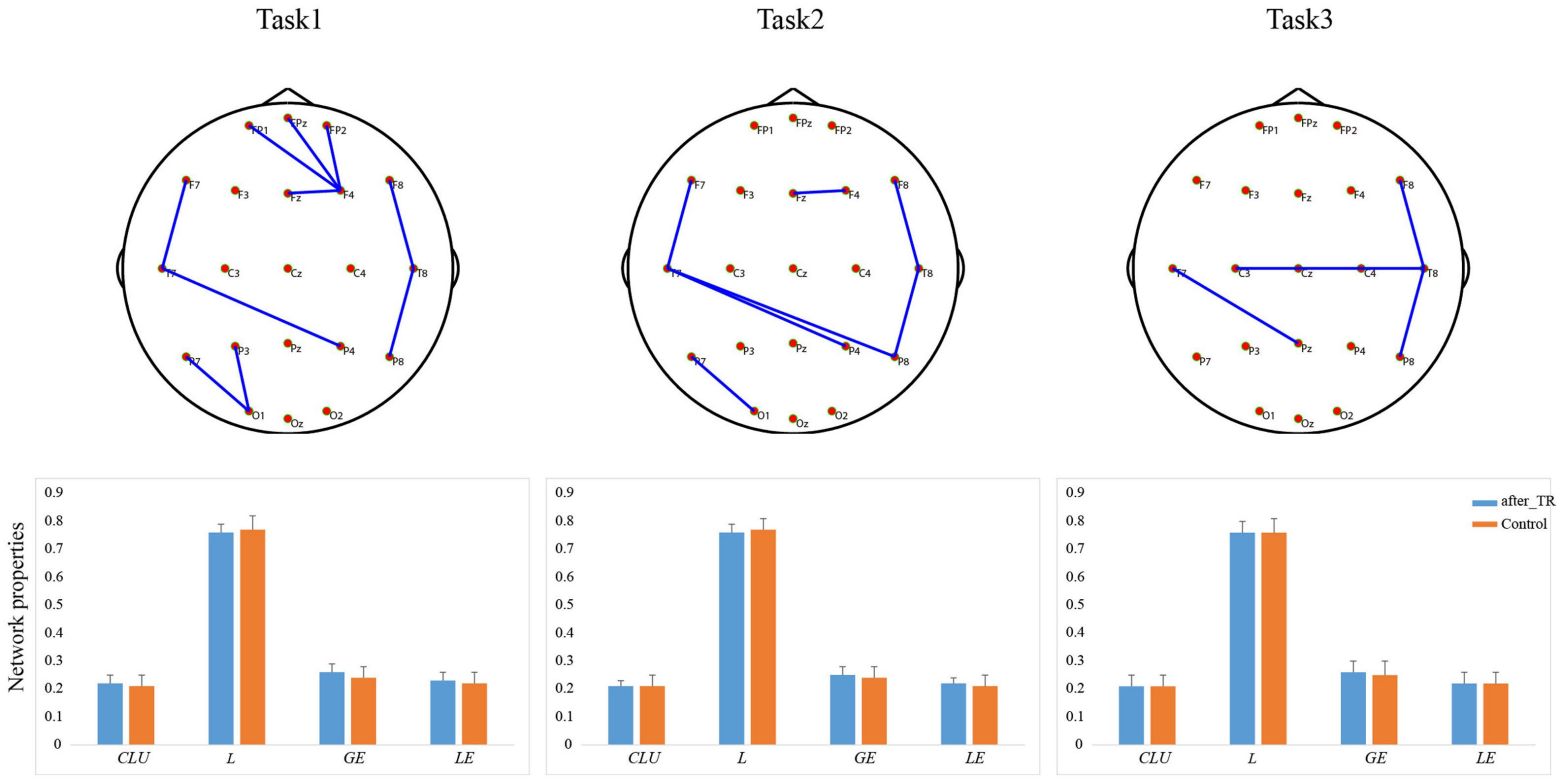
The first line is network topology differences between the after_TR group and the control group in the cool executive tasks (Task 1: THT; Task 2: TMT-A; Task 3: TMT-B). The corresponding network properties in the three tasks were below the network topologies. The blue solid lines denote the stronger network edges (*p* < 0.05, FDR corrected) of the after_TR group compared to those of the control group.

In the THT, there was no significant difference between the two groups in the reaction time and the operative step (*p* > 0.05). In the TMT-A, we found that the reaction time of the after_TR group was much longer (*p* < 0.05) than that of the HC group, while there was no significant difference in the error number between the two groups. In the TMT-B, compared to the control group, the after_TR group showed longer reaction time and more error numbers (*p* < 0.01). We also explored the underlying network differences between the after_TR group and the control group in the cool executive tasks (THT; TMT-A; TMT-B). In Figure 5, there is no significant network topology difference between the after_TR group and the HC group in the cool executive tasks, and no significant differences in the network properties were found (*p* > 0.05).

### 3.4. The clinical efficacy prediction based on the change in network properties

**Figure 6:**
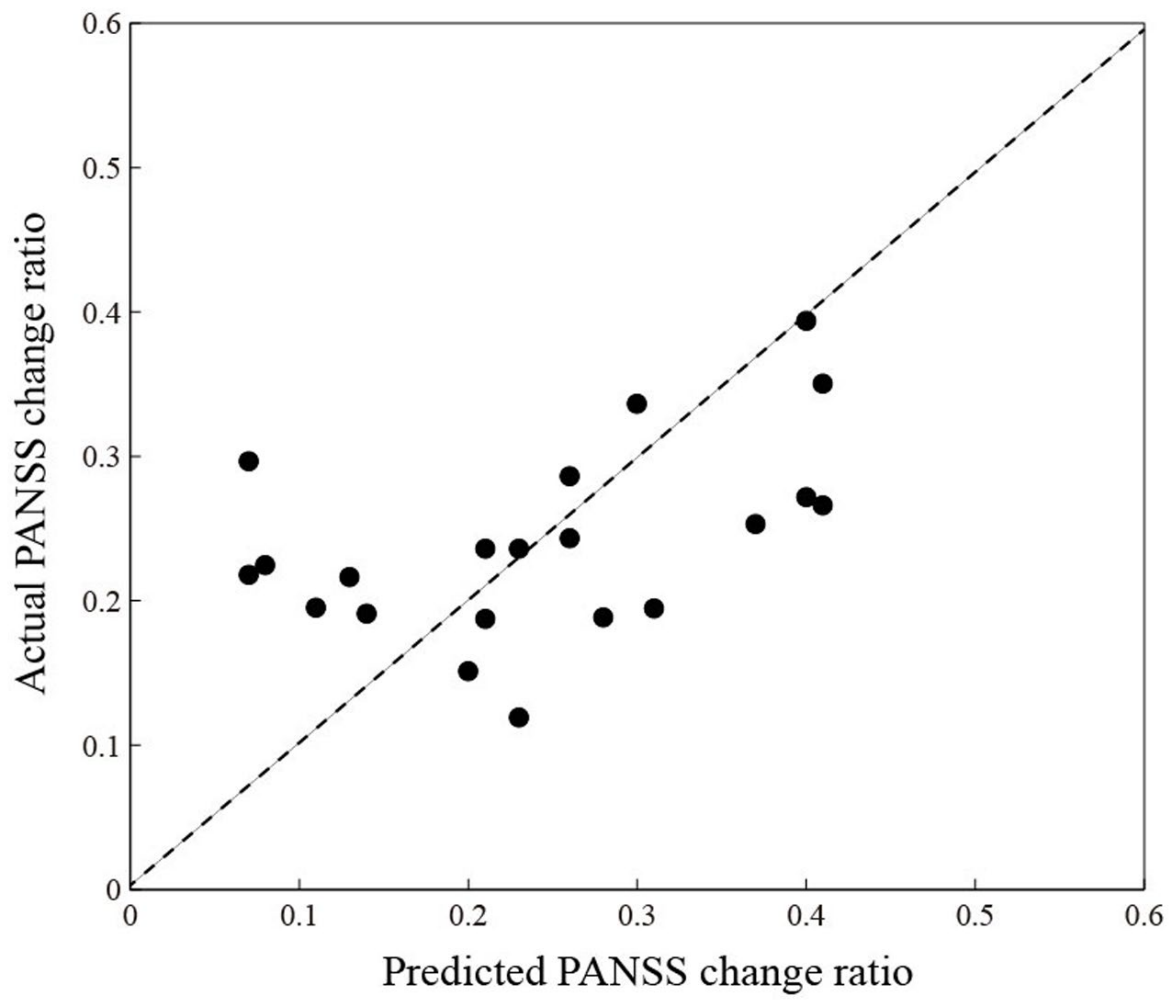
The correlation between predicted (*X*-axis) and actual (*Y*-axis) PANSS change ratios. The black-filled circles are individuals living with schizophrenia.

Given the relationship between the change network properties and the PANSS change ratio (*p* < 0.05), the change network properties (*CLU*, *LE*, *GE,* and *L*) in the cool executive tasks may be features to predict the patient’s PANSS change ratio. Figure 6 shows the correlation between predicted and actual PANSS change ratios, where the *X* and *Y* axes illustrate the predicted and actual PANSS change ratios, respectively. Pearson’s correlation coefficient between predicted and actual PANSS change ratios was *r* = 0.48 (*p* = 0.03), and the corresponding root means square error was 9.90%. Based on the 21 patients with SP, we finally built the regression model to predict the clinical efficacy, and the model was *Y* = 0.197–2.081*L.

## 4. Discussion

The cool executive function may be a key source of skills in life and occupation, and have critical impacts on human quality of life (24), and cool executive function recovery in people living with SP may help them to better seamlessly integrate into society and work. The present study investigated the changes in behavioral performance and brain networks under the cool executive tasks in patients with SP after atypical antipsychotic treatment for 8 weeks. The behavioral data from the TMA-A and TMT-B tasks suggested that the before_TR group overall showed a longer reaction time than that of the HC group, reflecting slower processing speeds in patients with SP to complete relevant cool executive operations, which is consistent with preceding studies (25, 26). A recent study by Ojeda et al. utilized confirmatory factor analysis to evaluate the fit of the 6-factor model and found that patients with SP showed a slowing processing speed in the neuropsychological measures of working memory and executive functioning (27). The slower processing speed in individuals living with SP may be related to the inability to attenuate the perception effect of unimportant information, and the tendency to make irrelevant observations (28). Although the error number in the TMT-A and the operative step in the THT related to a series of cognitive processes flexibly in problem-solving, the present study revealed that there was no significant difference between the patients with SP and the healthy controls. Given that TMT-A and THT with 3-level only involve the simple cool executive operation and maybe do not require much cognitive resources and energy compared to complex tasks, patients with SP can solve problems and attain goals smoothly. Frith et al. adopted a simple motor task that is designed to elicit errors to explore the problem-solving ability of patients with SP and found healthy controls and patients could correct the existing errors in the absence of visual feedback (29). Nevertheless, in the context of a more complex executive task (TMT-B), the cognitive impairment in patients with SP was highlighted by a slower response and more errors and could not flexibly solve the existing problems well. Emerging evidence has denoted that to accomplish a complex executive task and achieve its required goals individuals need to implement a successful cognitive operation which is required multiple cognitive resources (i.e., attention, working memory, and learning) working together (30, 31). For instance, one study utilized the WCST, which is involved in constant cues and underlying principles as well as feedback regarding prior responses, revealed that patients with SP performed worse with more errors and fewer categories compared to the controls after controlling the age, sex, and education (32). As revealed by the current study, in the TMT-B, which requires flexible attention set-shifting between the numbers and the letters, the before_TR group showed more error numbers than those of the control group. Clinically, we found there was a positive relationship between reaction time in TMT-A and the patients’ positive symptoms. The main positive symptoms of schizophrenia patients are abnormal thinking, delusions, and auditory hallucinations (33).

Although there is a gap in the cool executive performance between the before_TR group and the HC group, we further presented that the cool executive function of the after_TR group was improved relative to the before_TR group through the atypical antipsychotic treatment for 8 weeks. The reaction time of the after_TR group was much shorter than that of the before_TR group in the TMT-A and TMT-B. What’s more, the after_TR group showed fewer error numbers in the TMT-B than those of the before_TR group. It reflected that the processing speed and problem-solving abilities in patients with SP were improved, which indicated that the cool executive dysfunction of SP could be improved by the atypical antipsychotic treatment. Multiple studies uncovered that the cognitive function (i.e., planning, sequencing, and executive function) in patients with SP improved and performed better in cognitive tasks after the antipsychotic treatment (34, 35). For instance, in a systematic review, Houthoofd confirms the efficacy of the antipsychotic treatment in improving the reasoning and problem-solving skills that were evaluated by WCST in patients with SP (36). Moreover, the after_TR group showed lower scores in the PANSS than the before_TR group. This observation corroborated the findings achieved by Remberk et al. who observed that the clinical treatment may be more effective in improving cognitive function outcomes with a reduction of symptom severity and moderate some aspects of executive functions (35).

The human brain serves as a large-scale network, and efficient brain architecture, including multiple brain regions, engages in integrative accomplishment successfully during cognitive tasks (i.e., attention and decision-making) (37, 38). However, dysfunctions in individual-related regions disturbed the brain’s efficient processing of relevant information, which then led to cognition performance deficits (39). Moreover, the brain of patients with SP is also reshaped by this abnormality (40). The network topology difference in our present study consistently uncovered dysfunctions in the multiple brain areas in the before_TR group during the cool executive tasks. The DMN is a network that is characterized by being active at rest, but which is deactivated when a cognitive activity is performed in favor of alternative, more efficient networks under the task (e.g., on demand of an executive cognitive activity). Our results showed that during the cool executive tasks, there was no DMN activation in the HC group, but stronger DMN-like linkages were found in the before_TR group, which reflected the dysfunctional activation of the DMN in patients with SP. As illustrated by previous studies, schizophrenia patients showed increased sensitivity to self-referential thought and the external environment (41, 42), which may induce more dysfunctional brain network linkages. The brain regions of patients with SP were more activated than the controls, which resulted in increased functional linkage within the DMN. In addition, Koch et al. proposed the reduction of brain activation during the processing of decisions and learning made individuals more likely to make correct choices, whereas persons living with SP did not present this reduction in brain linkage, thus leading to impaired cognitive performance (43). After the atypical antipsychotic treatment for 8 weeks decreased DMN-like linkages were observed in patients with SP, which could be contributed to the effect of atypical antipsychotic treatment. Furthermore, there is a relationship between the changed network properties and the PANSS Change ratio in the current study, and the changed network properties (*CLU*, *LE*, *GE*, and *L*) may serve as the features to predict the patient’s PANSS change ratio.

There are some unanswered questions in the current study that need further inquiry. A limitation is that there were likely noises in the global network involving primary motor activity (beyond executive functions) that occurred during the tasks and the acquisition of the EEG signals (i.e., hand and pencil movement). Further, we will try to use executive function tests of simple motor response (e.g., single mouse-click selection response paradigms) to reduce these noises. The variability of the drug treatment (3 different types without enough separate samples to be tested) and the small sample size also need to further improve in this study. The assessment of the cool executive function only included THT and TMT A-B, we will adopt more comprehensive tasks to evaluate individuals’ cool executive function in our future work.

The present study revealed the behavioral and brain network changes of the cool executive function in individuals living with SP before and after atypical antipsychotic treatment, deepened our understanding of cool executive function in individuals living with SP, and might provide physiological information to reliably predict the clinical efficacy of the treatment. Future research should aim to parse out the intervention of cool executive tasks combined with atypical antipsychotic treatment. While the goal of the intervention of cognitive tasks is to be utilized in daily life, more work needs to be done for individuals living with SP thus promoting the transference of these cognitive benefits to daily function.

## Data availability statement

The raw data supporting the conclusions of this article will be made available by the authors, without undue reservation.

## Ethics statement

The studies involving human participants were reviewed and approved by the Ethics Committee of Xinxiang Medical University. The patients/participants provided their written informed consent to participate in this study.

## Author contributions

YS and HZ wrote and revised the manuscript. All authors contributed to the current manuscript revision and approved the submitted version.

## Funding

This work was supported by NSFC-henan mutual funds (U1704190), one thousand Zhongyuan Talents (204200510020), the China Postdoctoral Science Foundation (2021M700701), and the Open Program of the Henan Biological psychiatry key Laboratory (ZDSYS2022D01).

## Conflict of interest

The authors declare that the research was conducted in the absence of any commercial or financial relationships that could be construed as a potential conflict of interest.

## Publisher’s note

All claims expressed in this article are solely those of the authors and do not necessarily represent those of their affiliated organizations, or those of the publisher, the editors and the reviewers. Any product that may be evaluated in this article, or claim that may be made by its manufacturer, is not guaranteed or endorsed by the publisher.

## Supplementary material

The Supplementary material for this article can be found online at: https://www.frontiersin.org/articles/10.3389/fpsyt.2023.1154011/full#supplementary-material

## Footnotes

^1^http://www.nitrc.org/projects/bct/

## References

[1] Green MF Horan WP Lee J. Nonsocial and social cognition in schizophrenia: current evidence and future directions. World Psychiatry. (2019) 18:146–61. doi: 10.1002/wps.20624, PMID: 31059632 PMC6502429

[2] Mak M Tyburski E Starkowska A Karabanowicz E Samochowiec J. The efficacy of computer-based cognitive training for executive dysfunction in schizophrenia. Psychiatry Res. (2019) 279:62–70. doi: 10.1016/j.psychres.2019.06.041, PMID: 31302353

[3] Friedman NP Miyake A Altamirano LJ Corley RP Young SE Rhea SA. Stability and change in executive function abilities from late adolescence to early adulthood: a longitudinal twin study. Dev Psychol. (2016) 52:326–40. doi: 10.1037/dev0000075, PMID: 26619323 PMC4821683

[4] Yi Y Yun Z Yajing S Qiongqiong R Ren W Changqin J. Estimation of the cool executive function using frontal electroencephalogram signals in first-episode schizophrenia patients. Biomed Eng Online. (2016) 15:131. doi: 10.1186/s12938-016-0282-y 27884145 PMC5123362

[5] Rodrigues CL Rocca CCD Serafim A Dos Santos B Asbahr FR. Impairment in planning tasks of children and adolescents with anxiety disorders. Psychiatry Res. (2019) 274:243–6. doi: 10.1016/j.psychres.2019.02.049, PMID: 30818146

[6] Knapp F Viechtbauer W Leonhart R Nitschke K Kaller CP. Planning performance in schizophrenia patients: a meta-analysis of the influence of task difficulty and clinical and sociodemographic variables. Psychol Med. (2017) 47:2002–16. doi: 10.1017/S0033291717000459, PMID: 28385166

[7] Mackenzie NE Kowalchuk C Agarwal SM Costa-Dookhan KA Caravaggio F Gerretsen P. Antipsychotics, metabolic adverse effects, and cognitive function in schizophrenia. Front Psych. (2018) 9:622. doi: 10.3389/fpsyt.2018.00622, PMID: 30568606 PMC6290646

[8] Vita A Gaebel W Mucci A. European Psychiatric Association guidance on treatment of cognitive impairment in schizophrenia. Eur Psychiatry. (2022) 65:e57. doi: 10.1192/j.eurpsy.2022.2315 36059103 PMC9532218

[9] Meltzer HY Mcgurk SR. The effects of clozapine, risperidone, and olanzapine on cognitive function in schizophrenia. Schizophr Bull. (1999) 25:233–56. doi: 10.1093/oxfordjournals.schbul.a033376 10416729

[10] Pardo BM Garolera M Ariza M Pareto D Salamero M Valles V. Improvement of cognitive flexibility and cingulate blood flow correlates after atypical antipsychotic treatment in drug-naive patients with first-episode schizophrenia. Psychiatry Res. (2011) 194:205–11. doi: 10.1016/j.pscychresns.2011.06.001, PMID: 22044531

[11] Goozee R Reinders AATS Handley R Marques T Taylor H Odaly O. Effects of aripiprazole and haloperidol on neural activation during the n-back in healthy individuals: a functional MRI study. Schizophr Res. (2016) 173:174–81. doi: 10.1016/j.schres.2015.02.023, PMID: 25778615

[12] Bruno A Pandolfo G Crucitti M Cedro C Zoccali RA Muscatello MRA. Bergamot polyphenolic fraction supplementation improves cognitive functioning in schizophrenia: data from an 8-week, open-label pilot study. J Clin Psychopharmacol. (2017) 37:468–71. doi: 10.1097/JCP.0000000000000730, PMID: 28591067

[13] Vita A Barlati S Ceraso A Nibbio G Ariu C Deste G. Effectiveness, Core elements, and moderators of response of cognitive remediation for schizophrenia: a systematic review and meta-analysis of randomized clinical trials. JAMA Psychiat. (2021) 78:848–58. doi: 10.1001/jamapsychiatry.2021.0620, PMID: 33877289 PMC8058696

[14] Lejeune JA Northrop A Kurtz MM. A meta-analysis of cognitive remediation for schizophrenia: efficacy and the role of participant and treatment factors. Schizophr Bull. (2021) 47:997–1006. doi: 10.1093/schbul/sbab022, PMID: 33772310 PMC8266668

[15] Fali L Lin J Yuanyuan L Yajing S Chanlin Y Yangsong Z. Brain variability in dynamic resting-state networks identified by fuzzy entropy: a scalp EEG study. J Neural Eng. (2021) 18:046097. doi: 10.1088/1741-2552/ac0d41 34153948

[16] Lin J Fali L Baodan C Chanlin Y Yueheng P Tao Z. The task-dependent modular covariance networks unveiled by multiple-way fusion-based analysis. Int J Neural Syst. (2022) 32:2250035. doi: 10.1142/S0129065722500356 35719086

[17] Peiyang L Huan L Yajing S Cunbo L Fali L Xuyang Z. EEG based emotion recognition by combining functional connectivity network and local activations. IEEE Trans Biomed Eng. (2019) 66:2869–81. doi: 10.1109/TBME.2019.2897651 30735981

[18] Fali L Yi L Luyan Z Chanlin Y Yuanyuan L Yuanling J. Transition of brain networks from an interictal to a preictal state preceding a seizure revealed by scalp EEG network analysis. Cogn Neurodyn. (2019) 13:175–81. doi: 10.1007/s11571-018-09517-6 30956721 PMC6426926

[19] Fali L Jiuju W Yuanling J Yajing S Wenjing P Limeng S. Top-down disconnectivity in schizophrenia during P300 tasks. Front Comput Neurosci. (2018) 12:33. doi: 10.3389/fncom.2018.00033 29875646 PMC5974256

[20] Kay SR Fiszbein A Opler LA. The positive and negative syndrome scale (PANSS) for schizophrenia. Schizophr Bull. (1987) 13:261–76. doi: 10.1093/schbul/13.2.261 3616518

[21] Fali L Chanlin Y Yuanling J Yuanyuan L Yajing S Jing D. Different contexts in the oddball paradigm induce distinct brain networks in generating the P300. Front Hum Neurosci. (2018) 12:520. doi: 10.3389/fnhum.2018.00520 30666193 PMC6330295

[22] Peng X Xiuchun X Qing X Peiyang L Rui Z Zhenyu W. Differentiating between psychogenic nonepileptic seizures and epilepsy based on common spatial pattern of weighted EEG resting networks. IEEE Trans Biomed Eng. (2014) 61:1747–55. doi: 10.1109/TBME.2014.2305159, PMID: 24845285

[23] Yin T Huiling Z Wei X Haiyong Z Yang L Shuxing Z. Spectral entropy can predict changes of working memory performance reduced by short-time training in the delayed-match-to-sample task. Front Hum Neurosci. (2017) 11:437. doi: 10.3389/fnhum.2017.00437 28912701 PMC5583228

[24] Fett AKJ Viechtbauer W Dominguez MD Penn DL Van Os J Krabbendam L. The relationship between neurocognition and social cognition with functional outcomes in schizophrenia: a meta-analysis. Neurosci Biobehav Rev. (2011) 35:573–88. doi: 10.1016/j.neubiorev.2010.07.001, PMID: 20620163

[25] Karbasforoushan H Duffy B Blackford JU Woodward ND. Processing speed impairment in schizophrenia is mediated by white matter integrity. Psychol Med. (2015) 45:109–20. doi: 10.1017/S0033291714001111, PMID: 25066842 PMC5297385

[26] Rodr Guez-S Nchez JM Crespo-Facorro B Gonz Lez-Blanch C Perez-Iglesias R Vazquez-Barquero JL. Cognitive dysfunction in first-episode psychosis: the processing speed hypothesis. Br J Psychiatry Suppl. (2007) 51:s107–10. doi: 10.1192/bjp.191.51.s107 18055925

[27] Ojeda N Pena J Schretlen DJ Sanchez P Aretouli E Elizagarate E. Hierarchical structure of the cognitive processes in schizophrenia: the fundamental role of processing speed. Schizophr Res. (2012) 135:72–8. doi: 10.1016/j.schres.2011.12.004, PMID: 22226902

[28] Fletcher PC Frith CD. Perceiving is believing: a Bayesian approach to explaining the positive symptoms of schizophrenia. Nat Rev Neurosci. (2009) 10:48–58. doi: 10.1038/nrn2536, PMID: 19050712

[29] Frith CD Done DJ. Experiences of alien control in schizophrenia reflect a disorder in the central monitoring of action. Psychol Med. (1989) 19:359–63. doi: 10.1017/S003329170001240X, PMID: 2762440

[30] Collins AGE Brown JK Gold JM Waltz JA Frank MJ. Working memory contributions to reinforcement learning impairments in schizophrenia. J Neurosci. (2014) 34:13747–56. doi: 10.1523/JNEUROSCI.0989-14.2014, PMID: 25297101 PMC4188972

[31] Moustafa AA Garami JK Mahlberg J Golembieski J Keri S Misiak B. Cognitive function in schizophrenia: conflicting findings and future directions. Rev Neurosci. (2016) 27:435–48. doi: 10.1515/revneuro-2015-0060, PMID: 26756090

[32] Zhiwei W Honghua Y Xuan W Hengyong G Meihong X Xiangyang Z. Interrelationships between oxidative stress, cytokines, and psychotic symptoms and executive functions in patients with chronic schizophrenia. Psychosom Med. (2021) 83:485–91. doi: 10.1097/PSY.0000000000000931 34080586

[33] Mclean BF Mattiske JK Balzan RP. Association of the jumping to conclusions and evidence integration biases with delusions in psychosis: a detailed meta-analysis. Schizophr Bull. (2017) 43:344–54. doi: 10.1093/schbul/sbw056, PMID: 27169465 PMC5605251

[34] Gallhofer B Bauer U Lis S Krieger S Gruppe H. Cognitive dysfunction in schizophrenia: comparison of treatment with atypical antipsychotic agents and conventional neuroleptic drugs. Eur Neuropsychopharmacol. (1996) 6 Suppl 2:S13–20. PMID: 8792116 10.1016/0924-977x(96)00010-7

[35] Remberk B Namysowska I Rybakowski F. Cognition and communication dysfunctions in early-onset schizophrenia: effect of risperidone. Prog Neuro-Psychopharmacol Biol Psychiatry. (2012) 39:348–54. doi: 10.1016/j.pnpbp.2012.07.007, PMID: 22819848

[36] Houthoofd SAMK Morrens M Sabbe BGC. Cognitive and psychomotor effects of risperidone in schizophrenia and schizoaffective disorder. Clin Ther. (2008) 30:1565–89. doi: 10.1016/j.clinthera.2008.09.014, PMID: 18840365

[37] Fali L Chanlin Y Yuanling J Yuanyuan L Yajing S Dezhong Y. The construction of large-scale cortical networks for P300 from scalp EEG. IEEE Access. (2018) 6:68498–506. doi: 10.1109/ACCESS.2018.2879487

[38] Yajing S Lin J Chanlin Y Qi Z Cunbo L Jing Y. The decision strategies of adolescents with different emotional stabilities in the unfair situations. Neurosci Bull. (2021) 37:1481–6. doi: 10.1007/s12264-021-00758-w 34378153 PMC8490599

[39] Fali L Jiuju W Yuanyuan L Chanlin Y Yuanling J Yajing S. Differentiation of schizophrenia by combining the spatial EEG brain network patterns of rest and task P300. IEEE Trans Neural Syst Rehabil Eng. (2019a) 27:594–602. doi: 10.1109/TNSRE.2019.2900725 30802869

[40] Junfeng S Yingying T Kelvin KOL Jijun W Shanbo T Hui L. Abnormal dynamics of EEG oscillations in schizophrenia patients on multiple time scales. IEEE Trans. Biomed. Eng. BME. (2014) 61:1756–64. doi: 10.1109/TBME.2014.2306424, PMID: 24845286

[41] Broyd SJ Demanuele C Debener S Helps SK James CJ Sonuga-Barke EJS. Default-mode brain dysfunction in mental disorders: a systematic review. Neurosci Biobehav Rev. (2009) 33:279–96. doi: 10.1016/j.neubiorev.2008.09.002 18824195

[42] Ongur D Lundy M Greenhouse I Shinn AK Renshaw PF. Default mode network abnormalities in bipolar disorder and schizophrenia. Psychiatry Res Neuroimaging. (2010) 183:59–68. doi: 10.1016/j.pscychresns.2010.04.008, PMID: 20553873 PMC2902695

[43] Kathrin K Claudia S Gerd W Julia S Christoph S Rgen J. Altered activation in association with reward-related trial-and-error learning in patients with schizophrenia. NeuroImage. (2010) 50:223–32. doi: 10.1016/j.neuroimage.2009.12.031 20006717

