# Supplementary material for: Antipsychotics-induced improvement of cool executive function in individuals living with schizophrenia
Source: Front Psychiatry. 2023 Apr 27;14:1154011. doi: 10.3389/fpsyt.2023.1154011 (PMC10172485; doi:10.3389/fpsyt.2023.1154011)
Supplement: Supplementary file 1 [file Data_Sheet_1.PDF]

In our current study, through the Cerebus 128TM Amplifier (Cyberkinetics In, American), the EEG dataset with 64 Ag/AgCl electrodes from the 10-20 system was acquired (Figure R2). The raw EEG was filtered with 0-250 Hz, and the sampling rate was 1000 Hz. The reference and ground electrodes were REF and GND, respectively, which could be found in Figure S1.

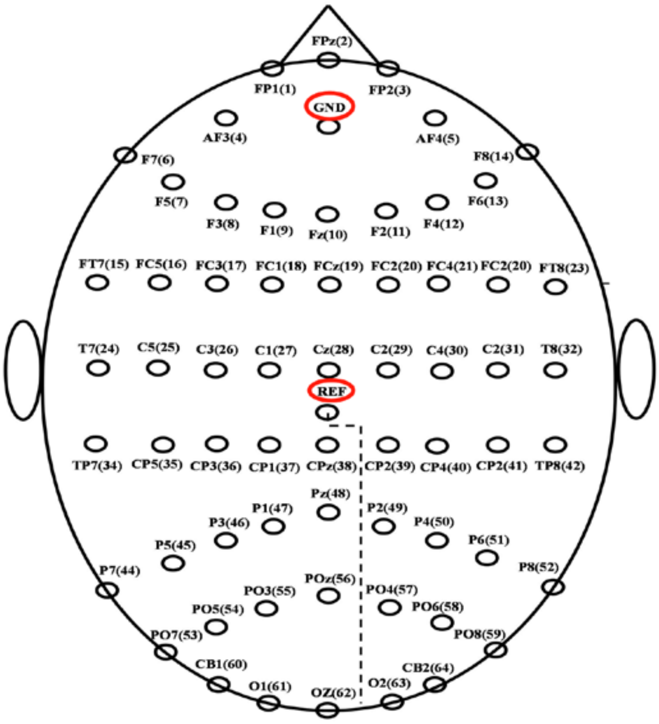

Figure S1. The position diagram with 64 Ag/AgCl electrodes.

Table S1. The brain network properties in the cool executive tasks for the before_TR, the after_TR, and the control groups (HC).

		Group		
		before_TR<br>（*n* = 21）	after_TR<br>（*n* =21）	HC<br>（*n* =24）
Task 1	*CLU*	0.309±0.081	0.220±0.031	0.214±0.044
	L	0.666±0.075	0.757±0.032	0.766±0.045
	GE	0.344±0.070	0.259±0.033	0.244±0.045
	LE	0.314±0.077	0.227±0.030	0.219±0.044

Task 2	*CLU*	0.307±0.083	0.214±0.024	0.210±0.038
	L	0.669±0.078	0.763±0.026	0.769±0.040
	GE	0.341±0.073	0.253±0.027	0.241±0.040
	LE	0.312±0.080	0.221±0.024	0.215±0.038
Task 3	*CLU*	0.300±0.079	0.213±0.037	0.214±0.044
	L	0.673±0.080	0.761±0.040	0.764±0.047
	GE	0.338±0.075	0.256±0.040	0.248±0.047
	LE	0.305±0.078	0.221±0.037	0.220±0.044

Note： Task 1: the Tower of Hanoi Task. Task 2: Trail-Marking Test A. Task 3: Trail-Marking Test B the reaction time.
